# Simple coiling using single or multiple catheters without balloons or stents in middle cerebral artery bifurcation aneurysms

**DOI:** 10.1007/s00234-012-1119-4

**Published:** 2012-11-29

**Authors:** Sung-Chul Jin, O-Ki Kwon, Chang Wan Oh, Jae Seung Bang, Gyojun Hwang, Nam Mi Park, Eun A Jung, Moon Hee Han, Hyun-Seung Kang, Hyun Park

**Affiliations:** 1 Department of Neurosurgery, Inje University, Haeundae Paik Hospital, Busan, South Korea; 2 Department of Neurosurgery, Seoul National University Bundang Hospital, 300 Gumi-dong, Bungdang-gu, Seongnam, Gyeonggi 463-707 South Korea; 3 Department of Radiology, Seoul National University Hospital, Seoul, South Korea; 4 Department of Neurosurgery, Seoul National University Hospital, Seoul, South Korea; 5 Department of Neurosurgery, Gyeongsang National University Hospital, Gyeongsang National University School of Medicine, Jinju, South Korea

**Keywords:** Aneurysm, Coiling, Simple, Middle cerebral artery, Endovascular

## Abstract

**Introduction:**

We evaluated the outcomes of middle cerebral artery bifurcation (MCAB) aneurysms treated with simple coiling using single or multiple catheters without stents or balloons.

**Methods:**

This study included 100 patients with 103 MCAB aneurysms who underwent a simple coiling procedure without the adjuvant use of stents or balloons. The angiographic clinical outcomes and recurrence of these aneurysms were evaluated.

**Results:**

Of the 103 aneurysms, 102 (99.0 %) aneurysms were successfully treated with simple coiling. One patient died from the consequences of a procedural aneurysm rupture. The treatment-associated permanent morbidity and mortality rates were 0 and 1.0 %, respectively. Post-coiling angiograms showed 28 complete occlusions (27.2 %), 60 neck remnants (58.3 %), and 14 partial occlusions (13.6 %). A follow-up angiography (median duration, 30 months; range, 3–73 months) was performed in 80 lesions. Recanalisation was found in 28 lesions (35.0 %), of which 6 were complete occlusions, 18 were neck remnants, and 4 were partial occlusions, as determined by post-coiling angiograms. Among these lesions, 14 major recurrences were retreated with coiling (*n* = 12) and clipping (*n* = 2) without complications. Age (odds ratio [OR], 0.93; 95 % confidence interval [CI], −0.11 to −0.01; *p* = 0.03), the presence of a rupture (OR, 3.89; 95 % CI, 0.12 to 2.60; *p* = 0.03), and a wide aneurysm neck (OR, 6.40; 95 % CI, 0.57 to 3.14; *p* = 0.005) were significantly associated with the aneurysm recurrence, as determined by multivariable analyses.

**Conclusion:**

Our study suggests that simple coiling of MCAB aneurysms is feasible and safe; however, it has limitations in durability, particularly in ruptured or wide-necked aneurysms and in young patients.

## Introduction

Surgical clipping is a feasible and durable treatment for middle cerebral artery bifurcation (MCAB) aneurysms. In contrast, endovascular coiling is not a primary option for MCAB aneurysm treatment due to its unfavourable anatomical properties such as wide necks and the incorporation of important vessels. Advanced adjuvant devices, such as stents or balloons, have been used successfully with favourable outcomes [1–3]. Most reports have demonstrated that balloon-assisted coiling does not pose as an additive risk of thromboembolism or aneurysm perforation [4–6]. However, a few studies have indicated that balloon-assisted coiling may result in increased thromboembolisms compared to the single catheter technique [7, 8]. There has been reluctance in using devices such as stents or balloons because of the potential risks associated with its use at distal cerebral vessels, which are distal to the bifurcation of the internal carotid artery (ICA). For many years, we have demonstrated that our coiling method for MCAB aneurysms is simple and conventional using single or multiple microcatheters; neither a stent nor balloons are applied.

In this report, we summarise the clinical and radiological outcomes of our study to evaluate the safety, durability, and effectiveness of simple coiling in MCAB aneurysms.

## Materials and methods

### Patients

We retrospectively assessed 100 patients with 103 MCAB aneurysms who underwent a detachable coil embolisation in our institution between August 2003 and December 2008. The study was approved by the Institutional Review Board.

In our practice, ruptured MCAB aneurysms are treated on an emergent basis. For unruptured MCAB aneurysms, treatment was recommended in patients with aneurysms larger than 5 mm and if the patient was 75 years old or younger. If the MCAB aneurysms were smaller than 2 mm, then an annual follow-up evaluation was recommended. For MCAB aneurysms with sizes between 2 and 5 mm, the decision for treatment was dependent on the morphology of the aneurysm, such as the daughter sac, multiplicity, previous history of subarachnoid haemorrhage (SAH), patient's age, patient's emotional status, and/or technical feasibility.

The clinical data were obtained from the patient's medical records. Using procedural records and images, the aneurysm characteristics, including the location of the aneurysm, size of the aneurysm (width and height), aneurysm neck diameter, vessel incorporation, and procedural details, including the immediate angiographic results and complications, were reviewed.

### Endovascular treatment

Coiling is the primary treatment option considered when a patient refuses surgery or displays poor medical conditions. Coiling is not attempted when the aneurysm height is less than 2 mm or when the major branch is incorporated into the aneurysm dome. During the coiling procedure, none of the patients were treated with stent- or balloon-assisted coiling. A simple coiling method using single or multiple microcatheters was performed. A multiple microcatheter technique was applied for aneurysms with broad necks or incorporated vessels, and this operational procedures have been previously described [9, 10]. All of the procedures were performed under general anaesthesia using a biplane system (Integris Allura, Phillips Medical Systems, Netherlands). For unruptured aneurysms, the patients received an intravenous bolus injection of 3,000-U heparin, and 1,000-U heparin was given every hour thereafter. Heparin was not given in cases of acute subarachnoid haemorrhage (SAH).

Angiographic findings, including the determination of aneurysm size, degree of occlusion, and recurrence, were interpreted by a neurointerventionist (S-C. J.) who was blinded to the clinical and procedural data. The aneurysm size was measured using three-dimensional angiographic images. The aneurysm width was determined by measuring the longest diameter of the fundus parallel to the axis of the aneurysm neck. The height was determined as the longest diameter of the fundus vertical to the axis of the aneurysm neck. The angiographic outcomes were classified as a complete occlusion, residual neck, or partial occlusion according to the Raymond scale [11]. The aneurysm recurrence was defined as a new opacification of the aneurysm sac or as an increase in the size of the residual neck or sac, which was further classified into two subcategories: minor recurrence, which did not require retreatment, or major recurrence, which required retreatment.

Follow-up imaging was performed. The time-of-flight and contrast-enhanced MR angiographies (MRA) were performed. A digital subtraction angiography (DSA) was performed when major recanalisation was suspected on the basis of the follow-up MRA studies. Retreatment was considered when the major recanalisation was confirmed on the DSA. A trained physician's assistant performed a clinical assessment on the patients at the time of discharge and at the outpatient clinic after discharge. The Modified Glasgow Outcome Scale (GOS) scores, which were categorised into good (GOS 4 to 5) and poor (GOS 1 to 3) functional outcomes, were used to evaluate the clinical outcomes [12].

### Statistical analysis

A statistical analysis was performed to evaluate the factors that could affect the aneurysm recurrence with the aid of the medical research collaborating centre. Nominal data were analysed using *χ* ^2^ or Fisher's exact test and the numerical data with Student's *t* test or Mann–Whitney's *U* test, when appropriate. A two-tailed *p* < 0.05 was defined as statistically significant. Multivariable analysis using a generalised estimation equation was performed to determine the independent factors of the aneurysm recurrence. Variables were selected for entry into the multivariable model on the basis of the results of the backward selection method. Using this approach, the model was fitted with all of the variables of interest (univariate analyses, *p* < 0.1). Next, the least significant variable was removed if it was not significant at the selected critical level. We continued by successively re-fitting the reduced models and applying the same rule until all of the remaining variables were statistically significant. The odds ratios and 95 % confidence intervals were then calculated.

## Results

### General characteristics

Of the analysed 100 patients with 103 MCAB aneurysms, 39 (39.0 %) patients were male. The median age was 60.5 years (mean ± SD, 58.6 ± 13.1 years; range, 17–83 years). Fifty-eight (58.0 %) patients presented with SAH. The Hunt and Hess grades of the patients were as follows: grade II in 35 patients (60.3 %), grade III in 9 patients (15.5 %), grade IV in 10 patients (17.2 %), and grade V in 4 patients (6.9 %). The Fisher grades of the patients included grade I in 3 patients (5.2 %), grade II in 6 patients (10.3 %), grade III in 23 patients (39.7 %), and grade IV in 26 patients (44.8 %). The mean height, width, and neck size of the aneurysms were 5.1 ± 2.8, 5.2 ± 2.7, and 3.5 ± 1.6 mm, respectively. A broad aneurysm neck larger than 4 mm was observed in 31 patients (30.1 %), and 43 aneurysms (41.7 %) had incorporated branch arteries (M2) around the aneurysm necks.

### Procedural outcome and complications

The single conventional catheter and multiple microcatheter techniques were used in 58 (56.3 %) and 45 (43.7 %) aneurysms, respectively. A successful coil embolisation was achieved in 102 (99.0 %) lesions: (1) a complete occlusion in 28 aneurysms (27.2 %), (2) neck remnant in 60 aneurysms (58.3 %), and (3) partial occlusion in 14 aneurysms (13.6 %).

Fifteen (14.6 %) procedure-related adverse events occurred, including a procedural aneurysmal rupture in four aneurysms (3.9 %) and thromboembolism in ten aneurysm (9.7 %) cases. For ten cases with thromboembolic complications, intra-arterial thrombolysis using urokinase, abciximab, or tirofiban was performed in seven cases. A partial or complete recanalisation was achieved in all of the lesions. Most of the complications did not result in a neurological deficit. A procedure-related mortality developed in one (1.0 %) lesion with a procedural rupture. This patient with Hunt and Hess grade IV SAH developed a contrast leakage during the coiling procedure. Further packing of the aneurysm resulted in a complete occlusion; however, the patient died of intractable increased intracranial pressure despite a decompressive craniectomy.

### Clinical outcomes

Of the 58 patients with acute SAHs, 42 patients (72.4 %) showed favourable outcomes (GOS score ≥4) at discharge. Nine (15.5 %) of the 58 patients with SAH continued to show a moderate or severe disability (GOS score >1 and ≤3) mainly due to an initially poor clinical status. Seven (12.1 %) patients died for the following reasons: (1) intractable intracranial pressure caused by an initial haemorrhage including a large cerebral haematoma (*n* = 5), (2) major infarction due to a severe vasospasm (*n* = 1), and (3) aneurysm rupture during the procedure (*n* = 1). Of the 42 patients with unruptured MCA bifurcation aneurysms, all of the patients remained clinically intact.

Eighty-four patients were evaluated for a median duration of 29.5 months (range, 3 to 78 months). In the SAH group, 40 patients (68.9 %) exhibited favourable clinical outcomes. Four patients' severe or moderate disability (GOS score 2 or 3) remained (*n* = 3) or was aggravated (*n* = 1). All of the 40 patients with unruptured MCA bifurcation aneurysms maintained good clinical outcomes.

An aneurysm rupture occurred in two patients after the initial procedure. Coiling was performed on one patient with an unruptured MCA aneurysm using the multiple catheter technique and resulted in a neck remnant on the final angiogram. The patient developed SAH 17 months after the initial coiling and then underwent re-coiling, which resulted in no further disabilities. Coiling was performed on the other patient with a ruptured MCA aneurysm using a single catheter technique, which resulted in a neck remnant on the final angiogram. The patient had suffered from SAH for 6 months after the initial coiling and then underwent re-coiling with moderate disability (GOS 3).

### Follow-up angiographic outcomes

A follow-up angiography was performed in 77 patients with 80 aneurysms (77.7 %). The follow-up loss was mainly due to the patients' grave conditions. The median time from the coiling procedure to the follow-up angiography was 30.0 months (range, 3 to 73 months).

A recurrence of the aneurysm neck or sac was found in 28 (35.0 %) lesions. Retreatment was performed in 14 lesions (17.5 %), and the median time until retreatment was 14 months (range, 2–63 months), (1) within 6 months after coiling (*n* = 3), (2) between 6 months and 1 year after coiling (*n* = 2), (3) between 1 and 2 years after coiling (*n* = 7) and (4) beyond 2 years after the procedure (*n* = 2). Two of the patients were retreated by surgical clipping.

The aneurysm recurrence was significantly different according to the size of the aneurysm neck (*p* = 0.019), patient's age (*p* = 0.036), and rupture (*p* = 0.001). However, the recurrence rate did not differ according to the coiling techniques, packing density of the coils, presence of vessel incorporation, initial angiographic result, or width or height of the aneurysm (Table 1). In a multivariable analysis, young age (OR, 0.93; 95 % CI, −0.11 to −0.01; *p* = 0.03) and aneurysms with a ruptured (OR, 3.89; CI, 0.12 to 2.60; *p* = 0.03) or wide neck (OR, 6.40; 95 % CI, 0.57 to 3.14; *p* = 0.005) were independently associated with recurrence (Table 2).

**Table 1:** Univariate analysis of recurrence in the 80 MCA bifurcation aneurysms followed

Characteristics	No recurrence	Recurrence	*p* value
Aneurysm height (mm)			0.099
<7	47	21	
≥7	5	7	
Aneurysm width (mm)			0.766
<7	43	22	
≥7	9	6	
Aneurysm neck (mm)			0.019
<4	42	15	
≥4	10	13	
Relationship with vessel			0.197
No incorporation	32	13	
Incorporation^a^	20	15	
Presence of rupture			0.001
Yes	20	22	
No	32	6	
Age (years)			0.036
<60	24	20	
≥60	28	8	
Embolisation technique			0.512
Single catheter technique	30	14	
Multiple catheter technique	22	14	
Angiographic results			0.439
Complete	16	6	
Incomplete	36	22	
Packing density of coils	29.9 ± 14.3	26.7 ± 9.7	0.428

^a^Incorporation means that vessel branches originate from the aneurysm

**Table 2:** Multivariable analysis using GEE for evaluating variables related with recurrence in the 80 MCA bifurcation aneurysms followed

Parameter	Estimate	Standard Error	OR	95 % CI		*p* value
Intercept	4.4336	1.7235		1.0555	7.8116	0.010
Age	−0.0579	0.0268	0.9333	−0.1104	−0.0054	0.031
Presence of rupture						
Non-rupture	−1.3591	0.631	1	−2.5959	−0.1223	0.031
Rupture	0	0	3.8927	0	0	.
Aneurysm neck (mm)						
<4	−1.8566	0.6563	1	−3.143	−0.5702	0.005
≥4	0	0	6.4019	0	0	.

*OR* odds ratio, *CI* confidential interval

## Discussion

In this study, most of the MCA bifurcation aneurysms were coiled without serious complication using the single or multiple catheter technique. This low procedural morbidity and mortality may be related to the indication we use for coiling. For broad-necked or vessel-incorporated aneurysms, a multiple catheter technique was useful. A procedural complication that resulted in mortality occurred in one patient with a ruptured aneurysm (1 %). There was no procedural morbidity or mortality for the unruptured lesions. In the current literature, the morbidity and mortality rates associated with surgical clipping were 4.2–12.9 and 6.5–10 %, respectively, for ruptured MCA aneurysms [13–15]. For unruptured lesions, the reported morbidity rates were approximately 3 % [16, 17]. Our study suggests that simple coiling using single or multiple catheters without the use of stents or balloons may be performed with acceptable risks for MCA bifurcation aneurysms with favourable configurations. However, our study also shows that simple coiling using single or multiple catheters for MCAB aneurysms has cardinal limitations in the degree of occlusion and durability.

Our results showed that a partial occlusion was 14.3 %, which may be higher compared to previous studies [1–3]. For ruptured aneurysms, a partial occlusion may have a risk of re-bleeding.

The recurrence rate in this study was 35 %. Half of the patients required retreatment, which included surgical clipping. In our study, the overall retreatment rate of the coiled aneurysms except that of MCAB aneurysms was approximately 7 % (unpublished data). For the MCAB aneurysms, the mean follow-up duration was 30.0 months; however, the recurrence rates could be higher with a longer follow-up evaluation period.

Thromboembolism (9.7 %) developed mostly in the ruptured aneurysms, and the rate of thromboembolism was higher compared to other studies [1–3]. The high thromboembolism observed in this study may be related to the absence of heparinisation in the coiling of the ruptured aneurysms. Recently, however, significant thromboembolism, which was required for IA thrombolysis, developed in less than 1 % of the patients (unpublished data) during the coiling of ruptured aneurysms. Thus, we suggested that early experience with coiling may contribute to the relatively high rate of thromboembolism.

In this study, we did not use stent- or balloon-assisted techniques. Several reports have previously demonstrated that stents or balloons may be successfully applied to treat MCA bifurcation aneurysms [3, 18–21]; however, we were very reluctant to use these devices because of concerns regarding the potential risks of unexpected adverse events such as thromboembolism or aneurysm perforation distal to the bifurcation of ICA. Surgical risks for MCAB aneurysms were not very high. Until now, there have been no data on the outcomes of stent-assisted coiling in MCAB aneurysms. Several reports have demonstrated that the procedural complication rates of the stent-assisted technique for MCAB aneurysms are not higher compared to simple coiling and showed favourable durability [22, 23]. As our study suggests, simple coiling using single or multiple catheters has a high recurrence rate in MCAB aneurysms. The stent-assisted technique may be an alternative option to enhance durability in the coiling of MCAB aneurysms.

Our multivariable analysis results demonstrate that age, rupture, and wide neck characteristics are significant predicting factors of aneurysm recurrence. Several other studies have also shown rupture and wide neck as predicting factors for recurrence [1, 24–26]. In our study, we also identified age as an influencing factor for MCA bifurcation aneurysm recurrence. Moreover, recurrence occurred more often in young patients. This result may be related to the decreased elasticity and compliance in the vascular wall with increasing age, both of which contribute in the prevention of recanalisation in the treatment of the aneurysm sac.

## Conclusions

Simple coiling without a stent or a balloon is a feasible treatment method for MCAB aneurysms. However, the recurrence rate was high. More than one third of the aneurysms recurred. Approximately half of the recurred aneurysms required retreatment. Our study suggests that simple coiling of MCA bifurcation aneurysms is feasible but that it has a limited durability.
